# Supplementary material for: Dynamic repression by BCL6 controls the genome-wide liver response to fasting and steatosis
Source: eLife. 2019 Apr 15;8:e43922. doi: 10.7554/eLife.43922 (PMC6464608; doi:10.7554/eLife.43922)
Supplement: Supplementary file 1. [file elife-43922-supp1.docx]

| **Regulatory Region** | **Forward Primer** | **Reverse Primer** |
| --- | --- | --- |
| *Abhd2* +37,143 | TCTTTGTGCCTTGTGGAGTT | CTGCAGCAAAGCAGGATTTC |
| *Acot2* -6081 | CCACAAGGGCACTTGATACT | AGAGTGGGCACCATTTCAG |
| *Acot3* +1140 | GGGGCGTAAGGAGAGTAAGG | TAAGCCCGTTAGCGTTTGTT |
| *Acot4* -5338 | GGTCCATGAAACATTTACTCTCTTAAC | CAGATCCCTGTTGACTCTTTCC |
| *Acot4* +4073 | CACATCTTGGGCCACTGATTA | GGACCTCATTTCCAGTGAATCT |
| *Angptl4* +2159 | CACCCATGTGGTTCCCATTA | AGACTCAGCCTAGCCAAGTA |
| *Bdh1* -22,923 | CATCTGAAGGCTGCTCTCAA | GTCAGGGTTACCCAACCATAAG |
| *Cd36* +12099 | GCTGAGAGCAGAATGACACTAT | TCTGGGTGAATAATCTTCCCTATTT |
| *Cyp4a31* +7668 | CATTTGAGGCAAGGTTCACAC | CCATTTGGCAACTGCAAGAG |
| *Ehhadh* +4817 | CCCATAGGGAGAAAGATCCTCTA | AACCATCGTACACAGGACAAG |
| *Fads2* -20,953 | AAGTGCCAACCTGGAAGAA | CAATCTCTGACCTCTGATCTCTATG |
| *Hmgcs2* -112 | AGAGCCAGCCAGCTAGATA | AGGTCTCAGAACAAGTCAAAGG |
| *Hmgcs2* -13115 | AAACTCCAAGAGCGGATTGT | GTCAGAGTTCCCAAGGAAAGTAG |
| *Idh2* -14,059 | CAGTGCTACACCAGCAATCA | GCTGGCTTGAATGTCCAAATC |
| *Por* +1532 | TGGAGCTATTGGTAGCTTTGG | CAACAGAGAATCGATGAGGGTAG |
