# Supplementary material for: Dynamic repression by BCL6 controls the genome-wide liver response to fasting and steatosis
Source: eLife. 2019 Apr 15;8:e43922. doi: 10.7554/eLife.43922 (PMC6464608; doi:10.7554/eLife.43922)
Supplement: Supplementary file 2. [file elife-43922-supp2.docx]

| **Gene name** | **Forward Primer** | **Reverse Primer** |
| --- | --- | --- |
| *36b4* | AGATGCAGCAGATCCGCA | GTTCTTGCCCATCAGCACC |
| *Abcd1* | GGCTGAGCCATAGGAAGTAAG | GGTTTCGGCAGGGATGTAA |
| *Abcd2* | CAAACCCTGACCAGTCTCTTAC | TAGCTGTCCGGATGAGAGTATAG |
| *Abhd2* | CCAGCAAACCGAGGGAAATA | GACACTGTGGCTTAGGAATGT |
| *Acadvl* | CTTTGCAGGGACTCAAGGAA | CAAGCGAGCATACTGGGTATTA |
| *Acnat2* | CGAGCAAAGGAGCAGAGATT | GGGAAGATTGTGATGGTGGT |
| *Acot1* | CAGCAGCTCCAGACTTTCTT | CCCAACCTCCAAACCATCATA |
| *Acot2* | GTTGGGAACACCATCTCCTAC | CGACATCCAAGAGACCATCTTT |
| *Acot3* | CACCGCTACCTGGGATGTAAT | CCTTCCAAGCCTCTTTCTAGT |
| *Acot4* | TACAGGCTCATGGAAAGGAAAG | TGGGCACATGGGAAAGTAAG |
| *Acss3* | CCAGGAAGCATTCAAGCATTTA | CCACTCGGGACATCACATATAG |
| *Aldh3a2* | CAAGTGCTGAGAGGATGTAGAC | ATGGCCAAGGAGCAGTTATC |
| *Bcl6* | GCTCGGAATGCTCCATACTT | TCGAAATGCAGGGCAATCT |
| *Bdh1* | CACTGTTCTAGCTCCTGTCTTC | CATCCCGCTGTCAGGTAAAT |
| *Cd36* | AGTAGAACCGGGCCACGTAGA | CGCCAACTCCCAGGTACAAT |
| *Cyp4a31* | GCAGGAGGCAGAAAGGATTAT | CAAATCGGGTCAGGCTTAGAG |
| *Ehhadh* | GGTCGTTGGAGTTCCTGTTGCT | TGGGCAAGCTTGGGACTGGC |
| *Fgf21* | CTACACAGATGACGACCAAGAC | CTTTGAGCTCCAGGAGACTTTC |
| *Hadh* | ATGCCCTTTAGCACAGTTGGA | GGGCCACTGTCCTTGGGTA |
| *Hmgcl* | AGATGGGTGTGAGTGTTGTG | ATGTAGACCAGGTCCTCAGTAG |
| *Hmgcs2* | CACATGAGACCCTCGATCTTTC | CACTGGCTTCTCTTTCGTGTAG |
| *Idh2* | AACACCGACGAGTCCATTTC | TCAAGTAGAGCGGCCATTTC |
| *Pparα* | ACAAGGCCTCAGGGTACCA | GCCGAAAGAAGCCCTTACAG |
| *Pparδ* | GCCTCGGGCTTCCACTAC | AGATCCGATCGCACTTCTCA |
| *Pparγ* | GAACCTGCATCTCCACCTTATT | TGGAAGCCTGATGCTTTATCC |
| *Ucp2* | ATGGTTGGTTTCAAGGCCACA | CGGTATCCAGAGGGAAAGTGAT |
| *Vldlr* | GTGACCACAGCAGTATCAGAAG | CTGCCATCACTAAGAGCAAGAA |
